# Effects of an outpatient pulmonary rehabilitation program on exercise capacity, muscle fatigue, dyspnea, and functional status in post-COVID-19 patients: non-randomized clinical trial

**DOI:** 10.3389/fresc.2026.1921478

**Published:** 2026-09-16

**Authors:** Miriã C. Oliveira, Paolo Capodaglio, Heren N. C. Paixão, Adriano L. Fonseca, Larissa R. Alves, Jairo B. S. Ribeiro Júnior, Rodrigo F. Oliveira, Tiago V. Fernandes, Daniel G. Marconi, Rodrigo A. C. Andraus, Deise A. A. P. Oliveira, Claudia S. Oliveira, Carlos H. M. Silva, Orlando A. Guedes, Wilson R. Freitas Júnior, Luis V. F. Oliveira

**Affiliations:** 1 Human Movement and Rehabilitation Graduate Programn (PPGMHR), Evangelical University of Goiás (UNIEVANGELICA), Anápolis, Brazil; 2 Research Laboratory for Biomechanics, Orthopaedic Rehabilitation Unit, Rehabilitation and Ergonomics, Ospedale San Giuseppe, Milan, Italy; 3 Physical and Rehabilitation Medicine Sector, Department of Biomedical, Surgical and Dental Sciences, University of Milan, Milan, Italy; 4 Health Sciences Graduate Program (PPGCS), Faculty of Medical Sciences (FCMSCSP), Irmandade da Santa Casa de Misericórdia de São Paulo, São Paulo, Brazil; 5 Department Rehabilitation, Barretos Cancer Hospital, Barretos, São Paulo, Brazil; 6 Dentistry Graduate Program (PPGO), Evangelical University of Goiás (UNIEVANGELICA), Anápolis, Brazil

**Keywords:** COVID-19, dyspnea, exercise capacity, fatigue, functional status, pulmonary rehabilitation

## Abstract

**Background:**

COVID-19 may lead to persistent sequelae that impair functional capacity, resulting in dyspnea, fatigue, and limitations in activities of daily living.

**Objective:**

To evaluate exercise capacity, fatigue, dyspnea, and functional status in patients following hospitalization for COVID-19 who underwent an outpatient pulmonary rehabilitation program (PRP).

**Methods:**

The study was approved by the Research Ethics Committee of UniEVANGÉLICA (approval number 4,296,707; September 24, 2020) and registered at ClinicalTrials.gov (Identifier: NCT04982042). This prospective single-arm non-randomized clinical trial included symptomatic individuals after COVID-19 infection. Exercise capacity was assessed using the Six-Minute Walk Test, muscle fatigue using the Fatigue Severity Scale (FSS), modified Medical Research Council (mMRC) Dyspnea Scale, and functional status using the Post-COVID-19 Functional Status Scale (PCFS). The PRP consisted of cardiorespiratory training and muscle strengthening exercises performed three times per week for six weeks.

**Results:**

A total of 54 patients were included (29 in the Ward Group and 25 in the ICU Group). After the PRP, 6MWT distance increased from 428.52 ± 104.5 m to 503.19 ± 98.04 m (*p* < 0.0001), mMRC dyspnea decreased from 3.50 ± 1.31 to 1.56 ± 0.60 (*p* < 0.0001), PCFS improved from 2.91 ± 0.59 to 0.63 ± 0.75 (*p* < 0.001), and FSS decreased from 4.68 ± 1.50 to 2.97 ± 1.55 (*p* < 0.001). The Ward Group showed greater unadjusted gains than the ICU Group; however, multivariable analysis identified lower baseline 6MWD as the only independent predictor of improvement (*β* = −0.547; *p* < 0.001), whereas ICU admission was not independently associated with the magnitude of gain (*p* = 0.246).

**Conclusion:**

The outpatient PRP was associated with significant improvements in exercise capacity, fatigue, dyspnea, and functional status. Lower baseline exercise capacity predicted greater improvement, regardless of ICU admission.

## 1 Introduction

The clinical course of SARS-CoV-2 infection is not limited to the acute phase and may compromise long-term functional recovery in affected individuals (1). Post-COVID-19 condition affects multiple organ systems, with exercise intolerance, muscle fatigue, and chronic dyspnea representing its most disabling manifestations (2, 3). Approximately 70% of COVID-19 survivors present a six-minute walk distance (6MWD) below predicted values up to one year after hospital discharge (4).

Exercise intolerance in post-COVID-19 condition is multifactorial and cannot be attributed solely to residual pulmonary damage; therefore, improvement of the acute illness does not necessarily guarantee restoration of pre-morbid functional capacity (5, 6). Cardiac and peripheral autonomic dysregulation contributes to a vicious cycle of impaired aerobic performance, characterized by reduced peripheral carbon dioxide production and an exaggerated hyperventilatory response during exercise (7). Furthermore, a persistent prothrombotic state associated with endothelial dysfunction and microthrombi within the muscular capillary bed compromises oxygen transport capacity during physical exertion, precipitating anaerobic metabolism and early-onset fatigue (8).

The severity of the initial clinical presentation during hospitalization directly influences the trajectory of post-COVID-19 recovery. Patients requiring intensive care, particularly those exposed to prolonged invasive mechanical ventilation, are at increased risk of intensive care unit-acquired weakness (ICU-AW), resulting from prolonged immobilization, deep sedation, and the use of neuromuscular blocking agents (9). This condition is characterized by alterations such as disuse-induced muscle atrophy, increased protein catabolism, and neuromuscular dysfunction, ultimately leading to reduced muscle strength and endurance (10). These mechanisms directly affect functional status and quality of life, decreasing independence in activities of daily living (ADLs) and perpetuating the cycle of physical inactivity and progressive deconditioning (11).

From a pulmonary perspective, impairments in alveolar-capillary diffusion, reduced lung compliance, and ventilation–perfusion mismatch may persist long after the acute phase of the disease (12). In addition, respiratory muscle weakness contributes to an increased ventilatory demand during physical exertion (13). At the peripheral level, mitochondrial dysfunction and impaired oxygen extraction by skeletal muscles promote the early onset of anaerobic metabolism, thereby exacerbating the perception of dyspnea and fatigue (14, 15).

Recent evidence has demonstrated that Pulmonary Rehabilitation (PR) has been shown to be an effective and safe intervention for the recovery of patients with post-COVID-19 condition (16). A clinical trial reported that pulmonary rehabilitation programs (PRPs) can significantly improve functional capacity, as evidenced by increased 6MWD, in addition to enhancing peripheral muscle strength and reducing muscle fatigue following the intervention (17). PR is recognized as a multidimensional intervention based on comprehensive individualized assessment, integrating aerobic and resistance exercise training, respiratory muscle training, breathing retraining strategies, and patient education. Its primary goals are to promote sustainable behavioral changes and optimize physiological responses to exercise (18).

Therefore, the implementation of an outpatient PRP for post-COVID-19 patients is justified based on the hypothesis that this intervention can provide significant benefits in both clinical and functional outcomes. Accordingly, the present study aimed to evaluate exercise capacity, muscle fatigue, dyspnea, and functional status in patients following hospitalization for COVID-19 who participated in an outpatient PRP.

## 2 Materials and methods

### 2.1 Study design

This was a prospective, consecutive, single-center, single-arm non-randomized clinical trial conducted at the Pulmonary Rehabilitation Laboratory of the Evangelical University of Goiás (UniEVANGÉLICA), involving individuals who presented persistent symptoms following COVID-19 infection. The study design followed the recommendations of the Transparent Reporting of Evaluations with Nonrandomized Designs (TREND) statement (19), as illustrated in Figure 1.

**Figure 1:**
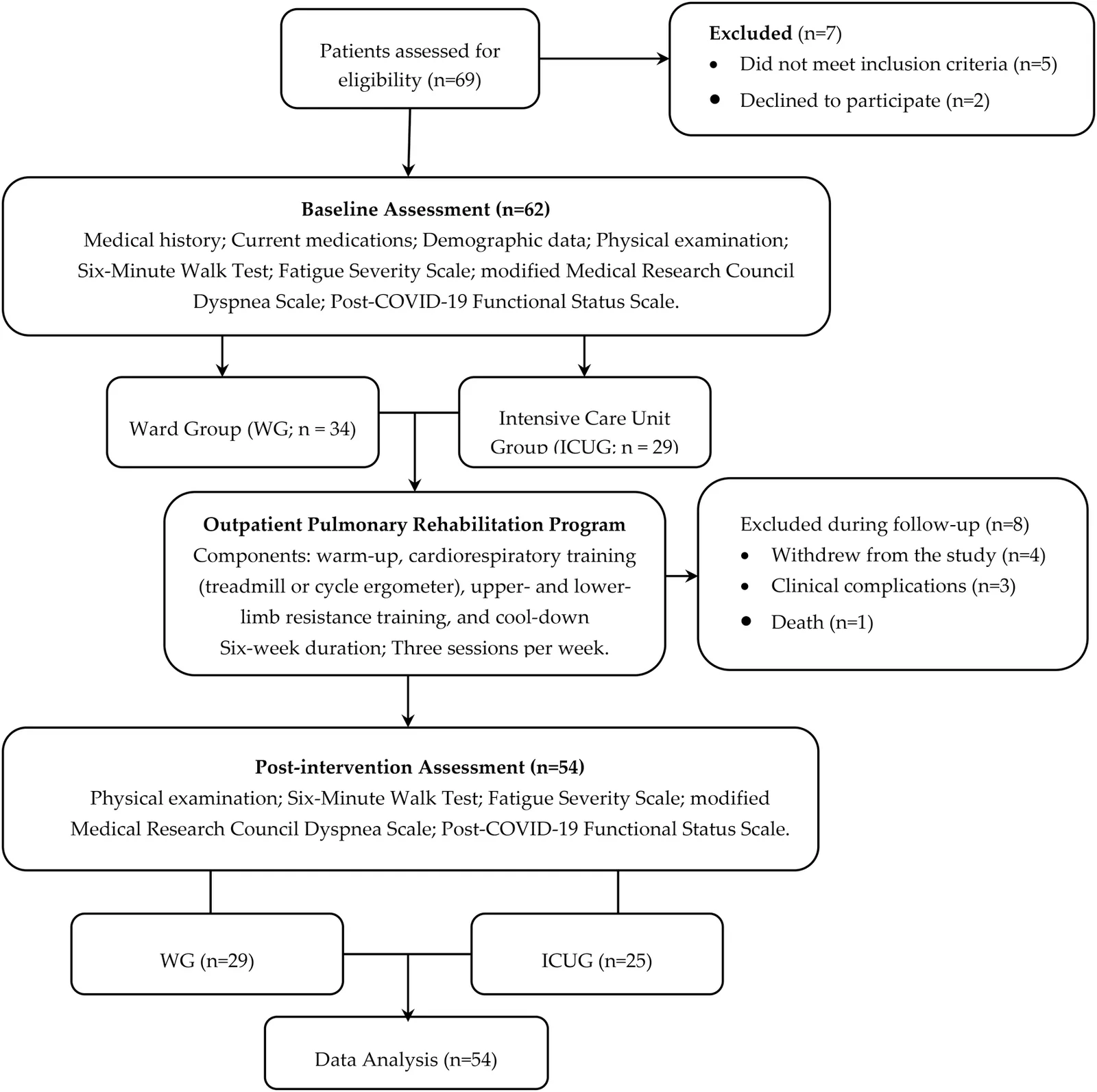
Flow diagram of participant recruitment, follow-up, and analysis.

### 2.2 Ethical considerations

The study was approved by the Research Ethics Committee of UniEVANGÉLICA (approval number 4,296,707; September 24, 2020) and registered at ClinicalTrials.gov (Identifier: NCT04982042). All participants provided written informed consent prior to enrollment and were informed of their right to withdraw from the study at any time without penalty. All study procedures were conducted in accordance with international biosafety guidelines for protection against COVID-19 transmission.

### 2.3 Participant recruitment

Participants were recruited between May 2021 and December 2021 through social media advertisements and banners distributed in referral hospitals for COVID-19 treatment, as well as among individuals seeking care through municipal and state healthcare services in the city of Anápolis, Goiás, Brazil.

### 2.4 Inclusion criteria

Adults of both sexes aged between 18 and 75 years were eligible for inclusion. Participants were required to be clinically stable and present persistent symptoms or sequelae following COVID-19 infection confirmed by reverse transcription polymerase chain reaction (RT-PCR), antigen testing, or serological testing, in addition to having a medical referral for pulmonary rehabilitation. Clinical stability was defined as maintenance of symptoms without the need for therapeutic modification and absence of antibiotic or corticosteroid use, except when prescribed as part of chronic treatment.

### 2.5 Exclusion criteria

Individuals were excluded if they had chronic neurological, psychiatric, or psychological disorders that could impair comprehension or participation in physical exercise; recent musculoskeletal disorders with incomplete recovery; uncontrolled systemic arterial hypertension (SAH) or unstable cardiovascular disease preventing exercise performance; or acute rheumatologic conditions.

### 2.6 Outcomes and assessment instruments

A multidisciplinary team of physicians and physiotherapists, previously trained for the study procedures, was responsible for patient assessment and monitoring throughout the outpatient PRP. Before enrollment, participants underwent a structured clinical evaluation, during which sociodemographic data, pre-existing comorbidities, acute COVID-19 complications, length of hospital stay and intensive care unit (ICU) admission, oxygen supplementation requirements, and ventilatory support modality were recorded.

Subsequently, exercise capacity testing and the administration of dyspnea, fatigue, and functional status questionnaires were performed by a physiotherapist. This procedure was performed at both assessments: before the start of the PRP (pre-PRP) and after completion of the six-week intervention (post-PRP).

#### 2.6.1 Exercise capacity—six-minute walk test (6MWT)

The 6MWT assesses the distance walked at a submaximal exercise intensity, defined as the highest level of exertion that can be continuously sustained for six minutes without transitioning to running. It is widely used as a simple, safe, and reproducible method for evaluating functional capacity and exercise tolerance (20). The test was performed according to the American Thoracic Society (ATS) guidelines (2002) (20) on a flat 30-meter corridor located in the UniEVANGÉLICA Sports Complex. Participants who discontinued the test before completion were excluded from the analysis. Vital signs (heart rate, peripheral oxygen saturation, and blood pressure) and nd ratings of perceived exertion (RPE) using the Borg scale were recorded immediately before the start (baseline) and immediately after completion (end of test) of the 6MWT. Predicted values were calculated using reference equations developed for the healthy Brazilian population (21).

#### 2.6.2 Muscle fatigue—fatigue severity scale (FSS)

The FSS evaluates fatigue severity based on its impact on motivation, physical activity, functional capacity, and activities of daily living (ADLs). It is a self-reported questionnaire consisting of nine items scored on a seven-point Likert scale, reflecting the participant's level of agreement with each statement. Participants were instructed to answer the questionnaire considering their experience during the previous week. The final score was calculated as the mean of the nine items. Higher grade indicate greater fatigue severity, and a score ≥ 4 was considered indicative of clinically significant fatigue (22).

#### 2.6.3 Dyspnea severity—modified medical research council (mMRC) dyspnea scale

The mMRC Dyspnea Scale is a widely used instrument for assessing the perception of breathlessness during activities of daily living. Owing to its simplicity and ease of administration, it is extensively employed in both clinical practice and research. The scale consists of five grades, from 0 to 4, with participants selecting the category that best reflects the degree of limitation imposed by dyspnea on their daily activities (23).

#### 2.6.4 Functional status—post-COVID-19 functional status (PCFS) scale

The PCFS Scale is a validated instrument used to assess functional limitations associated with COVID-19. The scale ranges from Grade 0 (no functional limitation) to Grade 5 (death). However, only Grades 0–4 were used in the present study, as Grade 5 was not applicable to the study population. Participants completed the questionnaire based on their health status during the seven days preceding the assessment (24, 25).

### 2.7 Interventions

Given that no specific recommendations for the management of COVID-19 were available during the pandemic period, the outpatient PRP implemented in this study was adapted from pulmonary rehabilitation recommendations proposed by the Global Initiative for Chronic Obstructive Lung Disease (GOLD) (26). The program lasted six weeks and consisted of three weekly sessions, each comprising four phases: warm-up, cardiorespiratory training, peripheral muscle strengthening, and cool-down/relaxation (Table 1).

**Table 1:** Cardiorespiratory and resistance training protocol implemented during the outpatient pulmonary rehabilitation program.

Training protocol implemented	Outpatient PRP	
	Weeks 1–3	Weeks 4–6
	Sets × Repetitions	Sets × Repetitions
Cardiorespiratory Training		
Treadmill or cycle ergometer		
Duration	30 min	40 min
Intensity (% of peak HR achieved during the 6MWT)	60%–70% (RPE 4–5)	70%–80% (RPE 5–6)
Resistance Training		
*Upper Limbs*		
Upper-limb diagonal pattern (D1, modified Kabat technique)^a^^,^^b^	3 × 10–12	3 × 13–15
Upper-limb diagonal pattern (D2, modified Kabat technique)^a^^,^^b^	3 × 10–12	3 × 13–15
*Lower Limbs*		
Hip flexion with knee extension	3 × 10–12	3 × 13–15
Extensores de joelho	3 × 10–12	3 × 13–15
Knee extension	3 × 10–12	3 × 13–15
Plantar flexion	3 × 10–12	3 × 13–15
Duration	20 min	30 min
Intensity (% of 1RM)	50%–60% (RPE 4–5)	50%–60% (RPE 5–6)

PRP, Pulmonary Rehabilitation Program; HR, heart rate; 6MWT, Six-Minute Walk Test; RPE, Rating of Perceived Exertion; D: diagonal; 1RM, one-repetition maximum; UL, upper limbs; LL, lower limbs.

a Adler SS, Beckers D, Buck M. PNF in practice: an illustrated guide. Springer Science & Business Media, 2007.

b Based on proprioceptive neuromuscular facilitation (PNF) diagonal movement patterns adapted from the Kabat method.

Progression of duration, aerobic intensity, and resistance load between phases was carried out provided that the participant demonstrated hemodynamic stability (systolic blood pressure <180 mmHg and diastolic blood pressure <110 mmHg, without a drop in systolic blood pressure of >20 mmHg compared to resting levels), SpO_2_ ≥ 88% during exercise, and a Borg score between 4 and 6. Patients who did not meet these criteria maintained the load from the previous week until they could progress safely.

At the beginning of each session, vital signs were assessed, followed by a warm-up consisting of alternating calisthenic exercises targeting the major muscle groups of both the upper and lower limbs, according to each participant's individual tolerance. Subsequently, cardiorespiratory training was performed following the FITT principle (frequency, intensity, time, and type). This approach was selected because of its ability to promote musculoskeletal and cardiovascular adaptations induced by physical exercise, while also contributing to the reduction of adverse respiratory symptoms (27).

Cardiorespiratory training was conducted using a continuous exercise model on either a treadmill or cycle ergometer, according to participant preference. The initial exercise duration was set at 30 min and was increased to 40 min after three weeks of participation in the PRP (18). Similarly, exercise intensity was initially prescribed at 60%–70% of the maximum heart rate achieved during the 6MWT and was progressively increased to 70%–80% during the final three weeks of the program (28).

A one-repetition maximum (1RM) test was performed to determine the maximum load that participants could lift in a single repetition while maintaining proper technique and without the ability to complete an additional repetition. Prior to testing, participants received detailed instructions and performed a familiarization set consisting of 20 repetitions of the exercise to be tested using light loads. This procedure was intended to familiarize participants with the movement pattern and provide adequate pre-test warm-up (29).

During the 1RM assessment, participants were instructed to avoid isometric contractions during the transition between the concentric and eccentric phases of the movement, as well as breath-holding associated with the Valsalva maneuver. The protocol included exercises targeting the major muscle groups of both upper and lower limbs. Testing began with a load below the estimated maximum and was progressively adjusted whenever the participant successfully completed the movement. Rest intervals of approximately five minutes were allowed between attempts to minimize short-term muscular overload. The test was terminated after a maximum of six attempts to reduce muscle fatigue and improve the reliability of the measurements (30).

Based on the results of the 1RM test, the initial resistance training load was prescribed at 50%–60% of the maximum load achieved and was progressively increased to 60%–70% after three weeks of training (31). Peripheral muscle strengthening aimed to improve muscle strength, hypertrophy, and neuromuscular efficiency, while optimizing the hemodynamic response to exercise and reducing energy expenditure during daily activities. Exercise prescription was based on fundamental training principles, including progressive overload, specificity, and periodization, as participants adapted to the training stimulus (32). The cool-down phase consisted of global stretching and relaxation exercises.

### 2.8 Safety assessment

All participants were supervised throughout the pulmonary rehabilitation program (PRP) by an experienced physiotherapist who was a member of the research team. Vital signs, including heart rate, peripheral oxygen saturation, and blood pressure, were monitored at the beginning, during, and at the end of each session and recorded on individual follow-up forms.

Peripheral oxygen saturation and heart rate were continuously monitored using an Oled Graph G-Tech pulse oximeter (Choice Electronic Technology Co., Ltd., Beijing, China). Blood pressure was measured using a Premium sphygmomanometer and stethoscope (Wenzhou Medical Instruments Co., Ltd., Ningbo, China) at the beginning and end of each session, or whenever the participant reported symptoms suggestive of blood pressure alterations.

RPE were assessed throughout cardiorespiratory training using the modified Borg scale for dyspnea and lower-limb fatigue. Whenever systolic and/or diastolic blood pressure exceeded predefined safety thresholds, participants were instructed to reduce exercise intensity or temporarily discontinue the activity until blood pressure values returned to acceptable levels. If clinical abnormalities persisted, the participant was referred for specialized medical evaluation.

### 2.9 Sample size calculation

The sample size calculation was based on the study by Hockele et al. (33), which used exercise capacity (6MWD) as the primary outcome. The calculation was performed using G*Power software (34, 35) for a paired-sample analysis, assuming an expected effect size of 0.41, 80% statistical power, and a significance level of 5% (*α* = 0.05). Accounting for an anticipated dropout rate of 10%, a total sample size of 51 participants was estimated to detect a statistically significant change.

### 2.10 Statistical analysis

The Kolmogorov–Smirnov test was used to assess the normality of data distribution. For pre–post comparisons, paired Student's *t*-tests were applied to variables with a parametric distribution, whereas the Wilcoxon signed-rank test was used for variables with a non-parametric distribution.

Comparisons between hospitalization groups (Ward vs. ICU) were performed using one-way analysis of variance (ANOVA), followed by Tukey's *post hoc* test when the null hypothesis was rejected. For variables with a non-parametric distribution, the Kruskal–Wallis test was employed, and when significant differences were detected, pairwise comparisons were conducted using the Mann–Whitney *U*-test.

To assess correlations between clinical and functional variables, Pearson's correlation coefficient was used for variables with a parametric distribution, whereas Spearman's rank correlation coefficient was applied to variables with a non-parametric distribution.

A multiple linear regression analysis was performed to identify factors independently associated with improvement in exercise capacity following the outpatient PRP. The dependent variable was the change in six-minute walk distance (Δ6MWD), calculated as post-PRP 6MWD minus pre-PRP 6MWD. Age, sex, body mass index (BMI), baseline 6MWD, length of hospital stay, and ICU admission were included as independent variables based on their clinical relevance. Sex was coded as female = 0 and male = 1, and ICU admission as no = 0 and yes = 1. Multicollinearity among independent variables was assessed using the variance inflation factor (VIF). Regression results are reported as unstandardized regression coefficients (B), standard errors (SE), standardized coefficients (*β*), 95% confidence intervals (CI), and *p*-values. Model performance was summarized using the coefficient of determination (R^2^), adjusted R^2^, and F statistic.

All statistical analyses were performed using the Statistical Package for the Social Sciences (SPSS) for Windows (Chicago, IL, USA), with the significance level set at *p* < 0.05.

## 3 Results

A total of 69 post-COVID-19 patients were assessed at admission to the outpatient PRP. Of the 62 patients initially enrolled in the study, 54 completed the outpatient PRP, yielding an adherence rate of 87%. For analysis, participants were stratified into two groups according to their hospital admission setting: (I) the Ward Group (WG), comprising 29 patients, and (II) the Intensive Care Unit Group (ICUG), comprising 25 patients.

Table 2 summarizes the demographic and clinical characteristics of the post-COVID-19 patients who participated in the PRP. The study population was predominantly male (62.96%) and of mixed race (40.74%). Participant age ranged from 24 to 77 years, with a mean age of 50.81 ± 12.41 years, while the mean body mass index (BMI) was 30.53 ± 6.28 kg/m^2^. No significant differences were observed between the WG and ICUG regarding sex, ethnicity, age, body weight, BMI, systolic blood pressure, or diastolic blood pressure. However, patients in the ICUG exhibited greater clinical severity, as evidenced by a longer length of hospital stay (19.00 ± 10.48 days vs. 10.59 ± 5.78 days; *p* < 0.0001), a higher requirement for invasive mechanical ventilation, and a longer interval between hospital discharge and initiation of the PRP (49.04 ± 29.10 days vs. 20.17 ± 19.79 days; *p* < 0.0001). The mean interval between hospital discharge and PRP admission for the total sample was 33.8 ± 28.8 days.

**Table 2:** Sociodemographic and clinical characteristics of post-COVID-19 patients enrolled in the outpatient pulmonary rehabilitation program.

Variables	Total (*n* = 54)	WG (*n* = 29)	ICUG (*n* = 25)	*p*-value
Sex				ns
Male	34 (62.96%)	16 (55.17%)	18 (72.0%)	
Female	20 (37.04%)	13 (44.83%)	7 (28.0%)	
Ethnicity				ns
White	21 (38.89%)	13 (44.83%)	8 (32.0%)	
Mixed race	22 (40.74%)	9 (31.03%)	13 (52.0%)	
Black	11 (20.37%)	7 (24.14%)	4 (16.0%)	
Age (years)	50.81 ± 12.41	51.97 ± 12.07	49.48 ± 12.91	ns
Body weight (kg)	84.64 ± 17.83	82.24 ± 15.32	87.43 ± 20.32	ns
BMI (kg/m^2^)	30.53 ± 6.28	29.89 ± 6.09	31.28 ± 6.53	ns
SBP (mmHg)	122.78 ± 14.06	123.10 ± 13.91	122.40 ± 14.51	ns
DBP (mmHg)	81.30 ± 9.72	80.69 ± 8.84	82.00 ± 10.80	ns
Length of hospital stay (days)	14.40 ± 9.19	10.59 ± 5.78	19.00 ± 10.48	*p* < 0.0001
ICU length of stay (days)	11.88 ± 8.63	N/A	11.88 ± 8.63	–
Ventilatory Support				*p* < 0.0001
Oxygen therapy	16 (29.63%)	13 (44.83%)	3 (12.0%)	
NIV	30 (55.56%)	16 (55.17%)	14 (56.0%)	
IMV	8 (14.81%)	N/A	8 (32.0%)	–
Endotracheal intubation	5 (9.26%)	N/A	5 (20.0%)	–
Tracheostomy	3 (5.56%)	N/A	3 (12.0%)	–
Time from hospital discharge to PRP admission (days)	33.82 ± 28.78	20.17 ± 19.79	49.04 ± 29.10	*p* < 0.0001

WG, Ward Group; ICUG, Intensive Care Unit Group; BMI, body mass index; SBP, systolic blood pressure; DBP, diastolic blood pressure; ICU, intensive care unit; NIV, non-invasive ventilation; IMV, invasive mechanical ventilation; PRP, pulmonary rehabilitation program; N/A, not applicable; ns, not significant.

Data are presented as mean ± standard deviation or *n* (%).

Patients in the ICUG exhibited greater clinical severity, as evidenced by a longer LOS (19.00 ± 10.48 days vs. 10.59 ± 5.78 days; *p* < 0.0001), a higher requirement for invasive mechanical ventilation, and a longer interval between hospital discharge and initiation of the PRP (49.04 ± 29.10 days vs. 20.17 ± 19.79 days; *p* < 0.0001). These findings suggest a greater degree of clinical and functional impairment among patients previously admitted to the intensive care unit, consistent with the increased severity of illness observed in this subgroup.

During the clinical interview, six participants (11.11%) reported temporary continuation of home oxygen therapy following hospital discharge. However, none of the patients required long-term oxygen therapy at the time of enrollment in the PRP. No statistically significant differences were observed between groups regarding sex, ethnicity, age, body weight, BMI, systolic blood pressure, or diastolic blood pressure, indicating baseline homogeneity for these variables.

The comorbidities reported by post-COVID-19 patients are presented in Table 3. The most prevalent conditions in the overall sample were SAH (38.89%), anxiety (25.93%), and obesity (24.07%). Although no statistically significant differences were observed between groups, a higher frequency of comorbidities was noted among patients in the ICUG compared with those in the WG.

**Table 3:** Pre-existing comorbidities reported at admission among patients who completed the outpatient pulmonary rehabilitation program.

Variables	Total (*n* = 54)	WG (*n* = 29)	ICUG (*n* = 25)	*p*-value
Anxiety	14 (25.93%)	5 (17.24%)	9 (36.0%)	ns
Asthma	2 (3.70%)	2 (6.90%)	0 (0.0%)	ns
Depression	3 (5.56%)	2 (6.90%)	1 (4.0%)	ns
Dyslipidemia	4 (7.41%)	2 (6.90%)	2 (8.0%)	ns
T2DM	7 (12.96%)	2 (6.90%)	5 (20.0%)	ns
COPD	1 (1.85%)	1 (3.45%)	0 (0.0%)	ns
Hepatic steatosis	3 (5.56%)	1 (3.45%)	2 (8.0%)	ns
AH	21 (38.89%)	10 (34.48%)	11 (44.0%)	ns
Hypothyroidism	2 (3.70%)	2 (6.90%)	0 (0.0%)	ns
Obesity	13 (24.07%)	5 (17.24%)	8 (32.0%)	ns

WG, Ward Group; ICUG, Intensive Care Unit Group; T2DM, type 2 diabetes mellitus; COPD, chronic obstructive pulmonary disease; SAH, systemic arterial hypertension; ns, not significant.

Data are presented as *n* (%).

During the acute phase of COVID-19, some patients developed clinical complications associated with the severity of the infection. In the WG, one patient (3.45%) presented acute myocardial infarction (AMI), one (3.45%) deep vein thrombosis (DVT), two (6.90%) pulmonary embolism (PE), and two (6.90%) renal complications. In the ICUG, one patient (4%) presented transient ischemic attack, one (4%) PE, and three (12%) renal complications. It is emphasized that, for the start of activities in the PRP, all these patients were already clinically stable, without the need for active therapeutic modification or hospitalization.

The exercise capacity of post-COVID-19 patients undergoing the outpatient PRP is presented in Table 4. The physiological and perceptual variables presented refer to measurements collected at the beginning (baseline) and at the end (end of test) of the 6MWT, assessed at two time points in the study: before the start of the program (pre-PRP) and after six weeks of rehabilitation (post-PRP).

**Table 4:** Changes in exercise capacity, physiological responses, and perceived exertion before and after the six-minute walk test, assessed at pre- and post-outpatient pulmonary rehabilitation program in post-COVID-19 Patients.

Variable	Total (*n* = 54)		WG (*n* = 29)		ICUG *(n* = 25)		*p*
	Pre-PRP	Post-PRP	Pre-PRP	Post-PRP	Pre-PRP	Post-PRP	
6MWD (m)	428,52 ± 104,5	503,19 ± 98,0^a^	433,4 ± 85,0	522,5 ± 93,9^b^	422,8 ± 125,0	480,76 ± 99,6^c^	*p* < 0.0001
Predicted 6MWD (%)	73,45 ± 15,9	87,85 ± 20,5^a^	75,3 ± 14,1	93,8 ± 23,0^b^	71,27 ± 17,9	80,93 ± 14,8^c^^,^^d^	*p* < 0.001
Baseline of 6MWT							
SBP (mmHg)	122,59 ± 10,7	120,19 ± 13,8	121,72 ± 10,0	120,00 ± 17,3	123,60 ± 11,5	120,40 ± 8,4	ns
DBP (mmHg)	81,67 ± 9,9	80,56 ± 8,6	80,34 ± 8,7	80,00 ± 9,3	83,20 ± 11,1	81,20 ± 7,8	ns
HR (bpm)	93,93 ± 16,3	85,38 ± 12,5^a^	90,72 ± 14,2	83,00 ± 10,9^b^	97,64 ± 18,1	88,25 ± 13,8^c^	*p* < 0.001
SpO_2_ (%)	95,43 ± 2,0	96,26 ± 1,5^a^	95,66 ± 1,7	96,59 ± 1,5^b^	95,16 ± 2,3	95,88 ± 1,4	*p* < 0.001
Borg Dyspnea Score	1,44 ± 2,3	0,35 ± 1,2^a^	1,31 ± 2,3	0,24 ± 0,8^b^	1,60 ± 2,2	0,48 ± 1,5^c^	*p* < 0.01
Borg LL Fatigue Score	2,19 ± 2,6	0,37 ± 1,1^a^	2,14 ± 2,5	0,28 ± 0,8^b^	2,24 ± 2,7	0,48 ± 1,3^c^	*p* < 0.001
End of 6MWT							
SBP (mmHg)	125,83 ± 11,8	124,44 ± 15,6	124,6 ± 11,3	125,2 ± 18,1	127,28 ± 12,4	123,60 ± 12,5	ns
DBP (mmHg)	83,15 ± 11,0	84,26 ± 9,6	82,1 ± 9,0	83,1 ± 9,7	84,40 ± 12,9	85,60 ± 9,6	ns
HR (bpm)	118,61 ± 19,5	116,47 ± 14,8	113,9 ± 16,1	113,9 ± 13,6	124,04 ± 22,0	119,38 ± 15,8	ns
SpO_2_ (%)	92,15 ± 3,6	93,61 ± 2,8^a^	92,6 ± 3,2	94,0 ± 3,0^b^	91,64 ± 4,1	93,20 ± 2,7^c^	*p* < 0.05
Borg Dyspnea Score	2,93 ± 2,9	0,98 ± 1,6^a^	3,0 ± 3,2	1,0 ± 1,5^b^	2,84 ± 2,6	0,96 ± 1,8^c^	*p* < 0.001
Borg LL Fatigue Score	4,39 ± 2,6	1,33 ± 1,8^a^	4,5 ± 2,7	1,2 ± 1,6^b^	4,24 ± 2,7	1,52 ± 2,0^c^	*p* < 0.0001
Peak HR (bpm)	125,64 ± 17,2	122,00 ± 12,8	121,5 ± 15,4	120,5 ± 10,8	130,52 ± 18,2	123,58 ± 14,7	ns
Lowest SpO_2_ (%)	89,93 ± 3,4	92,26 ± 2,9^a^	90,4 ± 3,7	92,2 ± 3,2^b^	89,37 ± 3,1	92,32 ± 2,9^c^	*p* < 0.001

WG, Ward Group; ICUG, Intensive Care Unit Group; PRP, Pulmonary Rehabilitation Program; 6MWD, six-minute walk distance; 6MWT, Six-Minute Walk Test; predicted (%), percentage of predicted value; SBP, systolic blood pressure; DBP, diastolic blood pressure; HR, heart rate; bpm, beats per minute; SpO₂, peripheral oxygen saturation; LL, lower limbs; RPE, Rating of Perceived Exertion; ns, not significant.

Data are presented as mean ± standard deviation.

a Statistically significant difference between pre- and post-PRP assessments in the overall sample.

b Statistically significant difference between pre- and post-PRP assessments in the WG.

c Statistically significant difference between pre- and post-PRP assessments in the ICUG.

d Statistically significant difference between WG and ICUG at the post-PRP assessment.

During the baseline 6MWT, 8 (14.81%) of the 54 participants were required to discontinue the test due to dyspnea, lower-limb muscle fatigue, or peripheral oxygen desaturation. Of these, 5 patients (17.24%) belonged to the WG and 3 patients (12.0%) to the ICUG. Following completion of the PRP, no participant required test interruption (*p* < 0.003).

A significant increase was observed in 6MWD (Δ = 74.67 m), as well as in the percentage of predicted values achieved (Δ = 14.4%), following the intervention (*p* < 0.0001). However, patients in the WG demonstrated greater functional improvement than those in the ICUG (*p* < 0.001).

Significant improvements were also observed in physiological and perceptual variables related to exercise performance, including reductions in resting heart rate (Δ = −8.55 bpm), increases in baseline peripheral oxygen saturation (SpO_2_) (Δ =  + 0.83%), reductions in baseline dyspnea perception (Δ = −1.09 points), and lower-limb muscle fatigue (Δ = −1.82 points). At the end of the 6MWT, improvements were also observed in final SpO₂ (Δ =  + 1.46%), dyspnea perception (Δ = −1.95 points), and lower-limb muscle fatigue (*Δ* = −3.06 points). Furthermore, minimum SpO_2_ recorded during the test increased significantly (Δ =  + 2.33%). Collectively, these findings demonstrate improved exercise tolerance and functional capacity following participation in the PRP among post-COVID-19 patients.

Table 5 presents the outcomes related to muscle fatigue, dyspnea perception, and functional status before and after participation in the outpatient PRP. A significant reduction in fatigue grade assessed by the Fatigue Severity Scale (FSS) was observed in both groups following the intervention (Δ = 1.71 points; *p* < 0.001), indicating a clinically meaningful improvement in perceived fatigue.

**Table 5:** Changes in fatigue severity, dyspnea severity, and functional status before and after the outpatient pulmonary rehabilitation program in post-COVID-19 Patients.

Variable	Total (*n* = 54)		WG (*n* = 29)		ICUG (*n* = 25)		*p*
	Pre-PRP	Post-PRP	Pre-PRP	Post-PRP	Pre-PRP	Post-PRP	
FSS	4,68 ± 1,50	2,97 ± 1,55^a^	4,56 ± 1,61	2,60 ± 1,34^b^	4,81 ± 1,38	3,37 ± 1,68^c^	*p* < 0.001
mMRC Dyspnea Score	3,50 ± 1,31	1,56 ± 0,60^a^	3,34 ± 1,34	1,55 ± 0,57^b^	3,68 ± 1,28	1,56 ± 0,65^c^	*p* < 0.0001
Grade 1	4 (7,41%)	27 (50%)	3 (10,34%)	14 (48,28%)	1 (4%)	13 (52%)	–
Grade 2	11 (20,37%)	24 (44,44%)	6 (20,69%)	14 (48,28%)	5 (20%)	10 (40%)	–
Grade 3	9 (16,67%)	3 (5,56%)	5 (17,24%)	1 (3,45%)	4 (16%)	2 (4%)	–
Grade 4	14 (25,93%)	0 (0%)	8 (27,59%0	0 (0%)	6 (24%)	0 (0%)	–
Grade 5	16 (29,63%)	0 (0%)	7 (24,14%)	0 (0%)	9 (36 %)	0 (0%)	–
PCFS	2,91 ± 0,59	0,63 ± 0,76^a^	2,69 ± 0,54	0,48 ± 0,74^b^	3,16 ± 0,55^c^	0,80 ± 0,76^d^	*p* < 0.001
Grade 0	0 (0%)	29 (53,70%)	0 (0%)	19 (65,52%)	0 (0%)	10 (40%)	–
Grade 1	0 (0%)	16 (29,63%)	0 (0%)	6 (20,69%)	0 (0%)	10 (40%)	–
Grade 2	12 (22,22%)	9 (16,67%)	10 (34,48%)	4 (13,79%)	2 (8%)	5 (20%)	–
Grade 3	35 (64,81%)	0 (0%)	18 (62,07%)	0 (0%)	17 (68%)	0 (0%)	–
Grade 4	7 (12,96%)	0 (0%)	1 (3,45%)	0 (0%)	6 (24%)	0 (0%)	–

PRP, Pulmonary Rehabilitation Program; WG, Ward Group; ICUG, Intensive Care Unit Group; FSS, Fatigue Severity Scale; mMRC, modified Medical Research Council; PCFS, Post-COVID-19 Functional Status Scale. ** *p* < 0.001; ** *p* < 0.001.

Data are presented as mean ± standard deviation or *n* (%).

a Statistically significant difference between pre- and post-PRP assessments in the overall sample.

b Statistically significant difference between pre- and post-PRP assessments in the WG.

c Statistically significant difference between the Ward Group and the ICUG at baseline (pre-PRP).

d Statistically significant difference between pre- and post-PRP assessments in the Intensive Care Unit Group (ICUG).

Regarding dyspnea, a significant reduction in mMRC dyspnea grade was observed after the intervention (Δ = 1.94 points; *p* < 0.0001). This improvement was accompanied by a marked decrease in the proportion of patients classified in the more severe dyspnea categories and a corresponding increase in the frequency of patients classified within the milder categories in both groups.

Functional status assessed using the Post-COVID-19 Functional Status (PCFS) Scale, also improved significantly following the PRP (Δ = 2.28 points; *p* < 0.001). This improvement was evidenced by an increase in the number of patients classified as Grade 0 or Grade 1 and a reduction in those classified as Grades 3 and 4. Patients in the ICUG exhibited greater functional impairment at baseline compared with those in the WG; however, both groups demonstrated significant functional recovery following the intervention.

The changes in exercise capacity, dyspnea, functional status, and muscle fatigue outcomes are also graphically presented in Figure 2. In all panels, significant reductions in post-PRP scores were observed in both the WG and the ICUG (*p* < 0.001 to *p* < 0.0001). Notably, the Ward Group demonstrated greater absolute gains in 6MWD compared to the ICU Group, although both groups showed statistically significant improvements across all evaluated outcomes.

**Figure 2:**
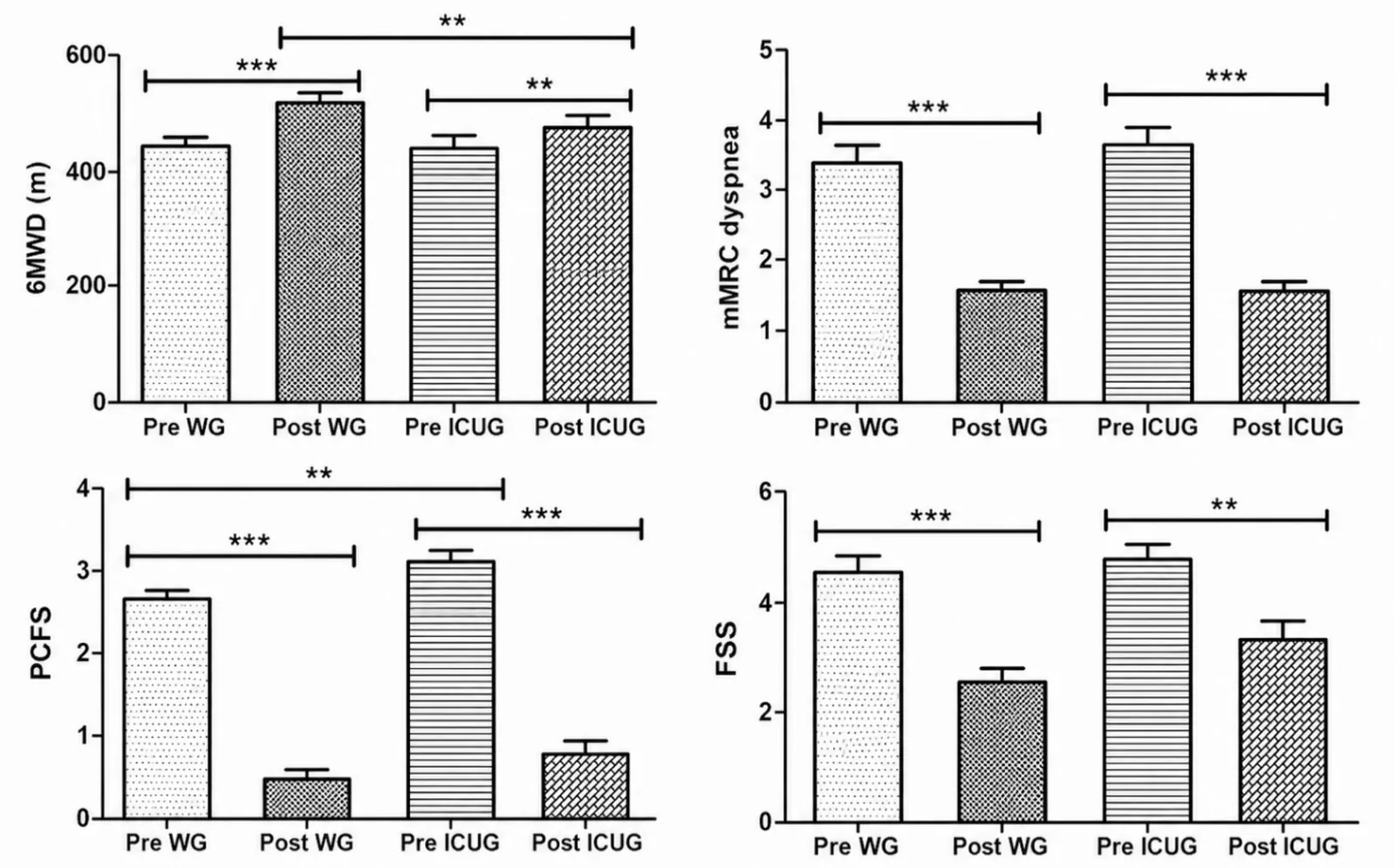
Exercise capacity, dyspnea perception, functional status, and muscle fatigue in post-COVID-19 patients undergoing the outpatient pulmonary rehabilitation program. WG, Ward Group; ICUG, Intensive Care Unit Group; 6MWD, six-minute walk distance; m: meters; mMRC, modified Medical Research Council; PCFS, Post-COVID-19 Functional Status Scale; FSS, Fatigue Severity Scale. **: *p* < 0.001; ***: *p* < 0.0001.

As shown in Figure 3, 6MWD was significantly and negatively correlated with age (*r* = −0.4040; *p* = 0.0024), length of hospital stays (*r* = −0.3975; *p* = 0.0042), PCFS score (*r* = −0.4816; *p* = 0.0002), and mMRC score (*r* = −0.3481; *p* = 0.0099). These findings indicate that older age, longer hospitalization, poorer functional status, and greater dyspnea severity were associated with lower functional exercise capacity.

**Figure 3:**
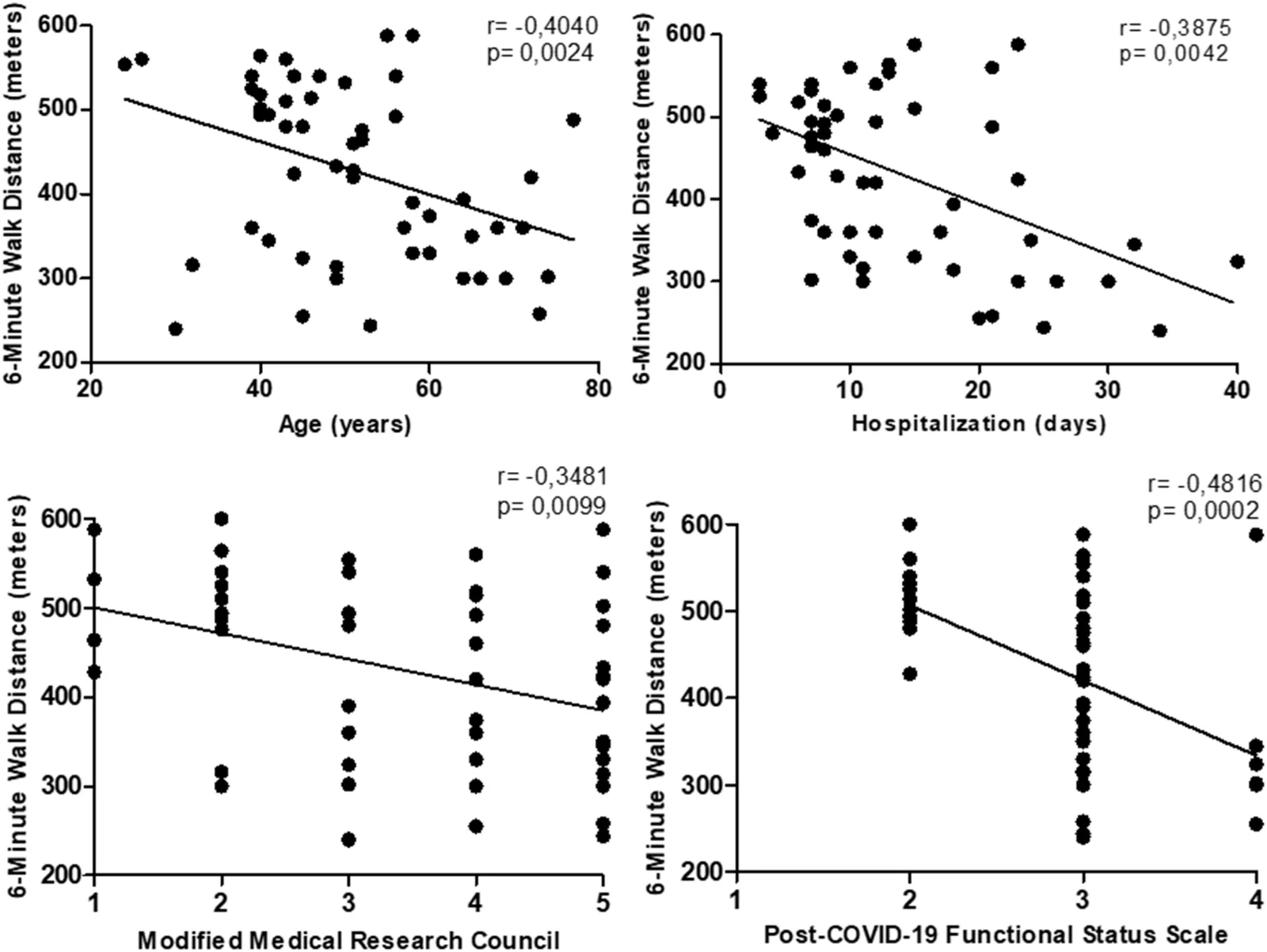
Correlations between Six-Minute walk distance and clinical and functional variables in post-COVID-19 patients.

As shown in Figure 4, significant positive correlations were observed between PCFS grade and age (*r* = 0.3314; *p* = 0.0144), length of hospital stay (*r* = 0.3321; *p* = 0.0151), and mMRC grade (*r* = 0.2826; *p* = 0.0384). Additionally, a significant positive correlation was found between FSS and mMRC grade (*r* = 0.3461; *p* = 0.0363). These findings indicate that greater dyspnea severity is associated with both increased functional limitation and higher levels of fatigue, whereas older age and prolonged hospitalization are associated with poorer post-COVID-19 functional status.

**Figure 4:**
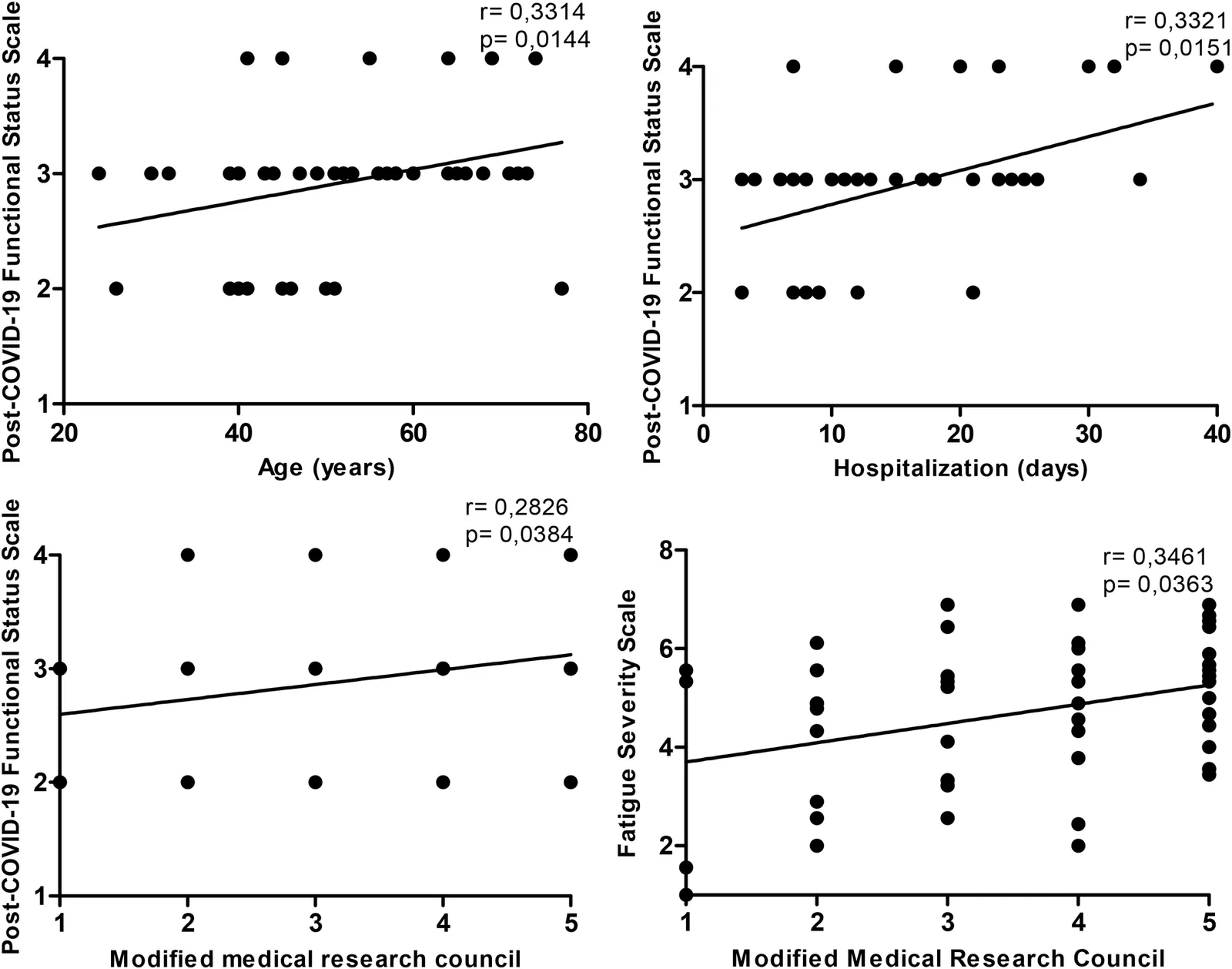
Correlations between functional Status, fatigue, dyspnea, and clinical variables in post-COVID-19 patients.

Multiple linear regression analysis was performed to identify factors independently associated with the magnitude of improvement in 6MWD following the outpatient PRP (Table 6). The overall regression model was statistically significant (F[6,47] = 3.627; *p* = 0.0049), explaining 31.6% of the variance in *Δ*6MWD (R^2^ = 0.316; adjusted R^2^ = 0.229). Baseline 6MWD was the only variable independently associated with Δ6MWD (B = −0.431; standardized *β* = −0.547; 95% CI: −0.632 to −0.230; *p* < 0.001), indicating that participants with lower baseline exercise capacity showed greater improvements in walking distance following the PRP. Age (*p* = 0.646), sex (*p* = 0.517), BMI (*p* = 0.735), length of hospital stay (*p* = 0.900), and ICU admission (*p* = 0.246) were not independently associated with *Δ*6MWD. Variance inflation factors ranged from 1.11 to 1.26, indicating no relevant multicollinearity among the independent variables.

**Table 6:** Multivariable linear regression analysis of predictors of improvement in six-minute walk distance following the outpatient pulmonary rehabilitation program.

Predictor	B	SE	Standardized *β*	95% CI for B	*p*-value
Age (years)	−0.384	0.830	−0.059	−2.054 to 1.287	0.646
Sex (male vs. female)	−14.503	22.202	−0.087	−59.167 to 30.162	0.517
BMI (kg/m²)	0.586	1.718	0.045	−2.871 to 4.043	0.735
Baseline 6MWD (m)	−0.431	0.100	−0.547	−0.632 to −0.230	<0.001
Length of hospital stay (days)	0.150	1.180	0.017	−2.225 to 2.524	0.900
ICU admission (yes vs. no)	−25.558	21.769	−0.158	−69.351 to 18.236	0.246

Model statistics: R^2^ = 0.316; adjusted R^2^ = 0.229; F(6,47) = 3.627; *p* = 0.0049; *n* = 54.

B, unstandardized regression coefficient; SE, standard error; β, standardized regression coefficient; CI, confidence interval; BMI, body mass index; 6MWD, six-minute walk distance; ICU, intensive care unit; PRP, pulmonary rehabilitation program.

The dependent variable was the change in six-minute walk distance (*Δ*6MWD), calculated as post-PRP 6MWD minus pre-PRP 6MWD. Sex was coded as female = 0 and male = 1; ICU admission was coded as no = 0 and yes = 1. Variance inflation factors ranged from 1.11 to 1.26, indicating no relevant multicollinearity among predictors. Bold indicates statistical significance (*p* < 0.05).

## 4 Discussion

The principal finding of this study was that a six-week outpatient PRP was associated with significant improvements in exercise capacity, reductions in dyspnea and fatigue, and substantial recovery of functional status in previously hospitalized post-COVID-19 patients. Although patients admitted to the intensive care unit exhibited greater baseline clinical impairment and longer hospital stays, both groups demonstrated favorable outcomes following the intervention, supporting the role of pulmonary rehabilitation in promoting functional recovery in this population (18, 36, 37).

Persistent manifestations following COVID-19 continue to represent a major challenge for healthcare systems, particularly among patients who required hospitalization during the acute phase of the disease. Dyspnea, fatigue, exercise intolerance, and functional limitations remain among the most frequently reported symptoms months after hospital discharge (38–40). Beyond physical impairments, recent evidence suggests that patients who required ventilatory support during hospitalization experience a substantial psychological burden and more complex recovery trajectories, indicating that post-COVID-19 recovery encompasses interconnected physical, emotional, and psychosocial dimensions (41, 42).

Regarding the recovery of exercise capacity, the findings of the present study demonstrated a significant increase in 6MWD after six weeks of pulmonary rehabilitation, consistent with previous studies that reported consistent improvements in exercise tolerance following structured rehabilitation programs in patients with post-COVID-19 syndrome (43–45). The mean increase of 74.67 m in the 6MWD observed in the total sample exceeds the 30 m threshold considered minimal clinically important difference (MCID) by the joint ATS/ERS consensus for chronic respiratory diseases (20), as well as the upper limit of 30.5 m identified in a systematic review of adults with various pathologies (46).

In this context, Gloeckl et al. (36), in a prospective cohort study involving 50 patients who underwent a three-week inpatient pulmonary rehabilitation program, reported increases of 48 m in the mild/moderate group and 124 m in the severe/critical group, both reaching statistical significance (*p* < 0.001). Although participants in the present study exhibited smaller absolute gains in the ICU group (+36.58 m) compared with the WG (+73.77 m), the relative improvement observed in the more severely affected group (+7.33% of predicted values) was lower than that reported by Gloeckl et al. This discrepancy may reflect differences in rehabilitation setting, duration, and training intensity, given the more intensive inpatient rehabilitation model employed in that study compared with the outpatient format adopted here.

Similarly, Hockele et al. (33), in a pilot clinical trial involving 29 post-COVID-19 patients with varying disease severity (mild to severe), demonstrated that a 16-session pulmonary and functional rehabilitation program (two months, twice weekly) produced a significant increase in 6MWD, from 326.3 ± 140.6 m to 445.4 ± 151.1 m (*p* < 0.001), with predicted values increasing from 59.7% to 82.6% (*p* < 0.001). In the present study, patients in the WG achieved improvements of comparable relative magnitude (+16.22% of predicted values), whereas the ICUG demonstrated a more modest increase (+7.33% of predicted values). These findings are consistent with the greater baseline functional impairment typically observed among patients who experienced more severe disease, as reported by Hockele et al.

Likewise, Oliveira et al. (44), in a meta-analysis of seven clinical trials (*n* = 188), reported a clinically meaningful increase in 6MWD of 60.56 m and a reduction in fatigue severity (FSS: −0.90), although no significant improvement in dyspnea was observed (mMRC: −0.57). In contrast, the present study demonstrated a substantial reduction in dyspnea severity (Δ = −1.86 in the WG and Δ = −2.12 in the ICUG; *p* < 0.0001). This discrepancy may be explained by the greater baseline severity of dyspnea among participants in the present cohort, who were predominantly classified within the highest mMRC categories, whereas baseline mMRC grade in the studies included in the meta-analysis were generally ≤1, thereby limiting the potential for detectable improvement. These findings support the hypothesis that multicomponent rehabilitation has been associated with adaptations across multiple functional domains, although the magnitude of the response may vary according to baseline disease severity and the extent of pre-existing functional impairment (47).

In the stratified analysis, patients previously admitted to the intensive care unit exhibited poorer baseline functional performance and smaller absolute gains compared with those admitted to hospital wards. These findings may reflect the greater clinical severity of patients requiring intensive care, including prolonged hospitalization, invasive mechanical ventilation, and extended periods of immobilization. Recent evidence indicates that factors associated with ICU admission may influence long-term outcomes and functional recovery (38, 48). Kolck et al. (49), using quantitative computed tomography in a cohort of 73 critically ill patients, demonstrated that individuals with COVID-19 experienced muscle mass loss at nearly twice the rate observed in other critically ill populations (2.1% vs. 1.2% per day; *p* = 0.008), with ICU length of stay identified as the only independent predictor of sarcopenia at three months. Such findings provide a plausible physiological basis for the greater baseline functional impairment observed among ICU survivors in the present study.

However, the multivariable analysis provides an important qualification to the between-group findings. After adjustment for age, sex, BMI, baseline 6MWD, length of hospital stay, and ICU admission, baseline 6MWD was the only variable independently associated with the magnitude of improvement in walking distance. The negative association between baseline 6MWD and *Δ*6MWD indicates that participants with poorer initial exercise capacity experienced greater gains following the PRP. This finding is consistent with previous rehabilitation studies showing substantial improvements in exercise capacity among post-COVID-19 patients with marked baseline functional impairment (33, 36, 45). In contrast, ICU admission was not independently associated with *Δ*6MWD, suggesting that the smaller unadjusted gains observed in the ICU group should not be interpreted as evidence of a lower responsiveness to pulmonary rehabilitation *per se*. Although ICU-related factors such as prolonged immobilization, mechanical ventilation, and accelerated muscle wasting may contribute to greater initial functional impairment and delayed recovery (9, 10, 49), the present adjusted analysis suggests that the magnitude of improvement in walking distance was more closely related to initial exercise capacity than to the hospitalization setting itself. This finding reinforces the importance of assessing baseline functional capacity when planning rehabilitation and interpreting treatment response in post-COVID-19 patients (18, 45).

The significant reduction in mMRC grade observed in the present study coincided with a meaningful improvement in dyspnea during activities of daily living, with most patients transitioning from more severe to milder dyspnea categories following pulmonary rehabilitation. Persistent post-COVID-19 dyspnea is recognized as a multifactorial phenomenon involving residual pulmonary abnormalities, cardiovascular dysfunction, physical deconditioning, and peripheral muscle impairment (50, 51). The mean reduction of ∼1.94 points on the mMRC scale exceeds the value of 1 unit recognized as clinically significant for this scale in patients with chronic respiratory diseases and post-COVID-19 (52). Santana et al. (50), through longitudinal cardiopulmonary phenotyping of COVID-19 survivors, demonstrated that persistent dyspnea may occur even in the absence of structural cardiopulmonary disease. The symptom was particularly pronounced among ICU survivors and appeared to be closely associated with physical deconditioning and post-intensive care syndrome (PICS).

This heterogeneity in recovery may also be mediated by alterations in cardiac autonomic regulation. Fonseca et al. (53) reported that post-COVID-19 patients presenting delayed heart rate recovery (≤12 beats/min) following the 6MWT exhibited poorer functional performance and greater dyspnea, independent of pulmonary function or maximal inspiratory pressure. These findings suggest that impaired vagal reactivation represents an additional mechanism contributing to exercise intolerance beyond purely pulmonary or muscular impairments. Within this context, aerobic exercise and resistance training may play a critical role in improving ventilatory efficiency and reducing the respiratory burden associated with physical activity (54).

The reduction in fatigue observed following pulmonary rehabilitation may be clinically relevant, as fatigue is recognized as one of the most disabling manifestations of post-COVID-19 syndrome (55, 56). Although a formal MCID for the FSS has not been established specifically for the post-COVID-19 population, estimates derived from studies in patients with chronic diseases indicate that a difference of at least 0.45 point on the FSS constitutes a clinically significant change (57). The mean reduction observed in the present study was 1.71 points, exceeding this threshold by more than threefold. Additionally, the sample mean shifted from 4.68 to 2.97, crossing the cutoff of ≥4 points recognized as indicative of clinically significant fatigue (22), which represents a qualitatively relevant change in the patients' fatigue status.

This finding is consistent with the recent meta-analysis of randomized controlled trials conducted by Pérez-Gisbert et al. (58), which demonstrated a moderate and statistically significant effect of pulmonary rehabilitation on fatigue reduction compared with control interventions (*p* = 0.0002), regardless of rehabilitation modality (supervised or home-based) or program duration (<12 or ≥12 weeks). These results further support the role of multicomponent rehabilitation strategies in alleviating fatigue and promoting functional recovery among post-COVID-19 patients (59, 60).

In contrast to the meta-analysis by Oliveira et al. (44), which did not identify a significant improvement in fatigue compared with controls, the present study demonstrated robust and consistent reductions in fatigue severity across both groups. This discrepancy may be attributable to the integrated multicomponent approach adopted in the present program (aerobic plus resistance training) and the greater baseline severity of participants, which provided a larger margin for detectable improvement.

Regarding functional status, the present findings revealed significant changes in PCFS grade in both study groups. A marked increase was observed in the proportion of patients classified within the lower grades of the scale following pulmonary rehabilitation, suggesting substantial functional recovery. Functional status represents a particularly relevant outcome in post-COVID-19 populations because it reflects the integrated impact of respiratory, muscular, and systemic manifestations on activities of daily living and social participation (61). A MCID has not yet been formally established for the PCFS (62); however, the mean transition of ∼2.3 functional categories observed in the present study, with a decrease from 77.8% to 0% in grades 3–4 and an increase from 0% to 83.3% in grades 0–1, indicates substantial functional recovery. This interpretation is supported by evidence that the PCFS is responsive to pulmonary rehabilitation in post-COVID-19 patients (24).

The correlation analyses further highlighted the interrelationship among the functional domains evaluated in this study. Reduced 6MWD was significantly associated with older age, longer LOS, greater dyspnea severity, and poorer functional status, findings that are consistent with previous evidence in the literature (63, 64). Older individuals appear to be more susceptible to the systemic consequences of COVID-19 infection, including accelerated muscle mass loss, reduced physiological reserve, and delayed functional recovery (65–67). Similarly, prolonged hospitalization predisposes patients to physical deconditioning, acquired muscle weakness, and persistent functional impairment following hospital discharge (49).

Furthermore, patients with more severe dyspnea simultaneously exhibited greater functional limitations and higher fatigue levels. These findings suggest that dyspnea may represent a central determinant in the persistence of post-COVID-19 functional impairment, directly affecting both exercise capacity and performance in activities of daily living (66, 67). Cecchetto et al. (68), in a multiparametric cardiopulmonary assessment of 50 post-COVID-19 patients with persistent dyspnea, reported that 62% presented diaphragmatic dysfunction, 48% exhibited hyperventilation, and 34% showed evidence of microvascular dysfunction, underscoring the heterogeneity of the pathophysiological mechanisms underlying this symptom. Likewise, Morgan et al. (69), in a systematic review of risk factors for persistent dyspnea, identified advanced age, prolonged hospitalization, and the need for mechanical ventilation as consistent predictors of chronic dyspnea, findings that closely align with the correlational results observed in the present study.

In summary, the beneficial effects observed following participation in the PRP may have been mediated by improvements in ventilatory efficiency and gas exchange, enhanced peripheral muscle oxidative capacity, and strengthening of both respiratory and locomotor muscles (70). Yang and Yang (71), in a review of pulmonary rehabilitation for the management of post-COVID-19 sequelae, reported that structured exercise training promotes cardiorespiratory adaptations that optimize pulmonary ventilation and tissue perfusion, mechanisms that are fundamental to reducing dyspnea and fatigue in post-COVID-19 patients. Collectively, these adaptations contribute to a reduced ventilatory burden during activities of daily living, improved exercise tolerance, and enhanced functional recovery (47).

Nevertheless, several limitations should be considered when interpreting the present findings. First, the absence of a control group precludes the isolation of the specific effects of the intervention from the natural course of recovery over time. This design choice was primarily driven by ethical considerations during the COVID-19 pandemic, as withholding pulmonary rehabilitation from symptomatic post-COVID-19 patients would have been inconsistent with emerging clinical recommendations. Furthermore, the differential response between the Ward and ICU groups suggests that baseline severity modulates rehabilitation outcomes, providing indirect evidence supportive of a treatment effect. Second, the single-center design and the follow-up period limited to the intervention phase restrict the generalizability of the findings to broader populations and healthcare settings.

Additionally, recruitment by medical referral and voluntary participation may have introduced a selection bias, as the included participants may represent individuals more motivated for functional recovery and with greater adherence to treatment. Furthermore, between-group comparisons and pre-post analyses for the remaining outcomes were not adjusted for baseline clinical severity, limiting the ability to distinguish between the effect of the hospitalization setting and the greater initial functional impairment of ICU patients. Finally, the lack of post-intervention follow-up prevents assessment of the long-term persistence of the observed benefits, an aspect of particular relevance given the chronic nature of post-COVID-19 condition.

Among the strengths of this study are its prospective design, the use of validated instruments for the multidimensional assessment of post-COVID-19 condition, and the inclusion of patients with heterogeneous levels of clinical severity, enabling evaluation of the impact of pulmonary rehabilitation across subgroups with distinct recovery profiles. Furthermore, the simultaneous assessment of exercise capacity, dyspnea, fatigue, and functional status provided a comprehensive understanding of the functional effects of the intervention in this population. Future multicenter randomized controlled trials incorporating control groups and long-term follow-up are warranted to confirm the sustainability of these benefits and to establish optimal pulmonary rehabilitation prescription parameters for post-COVID-19 patients.

## 5 Conclusions

A six-week outpatient PRP was associated with significant improvements in exercise capacity, dyspnea, fatigue, and functional status among previously hospitalized post-COVID-19 patients. Although greater unadjusted gains in exercise capacity were observed among patients previously admitted to hospital wards, ICU admission was not independently associated with the magnitude of improvement in 6MWD after adjustment for relevant demographic and clinical factors. Baseline 6MWD was the only variable independently associated with *Δ*6MWD, with lower initial exercise capacity associated with greater subsequent improvement. These findings highlight the importance of assessing baseline functional capacity when planning pulmonary rehabilitation and interpreting functional recovery in post-COVID-19 patients. However, given the single-arm design, these improvements should be interpreted as associations with participation in the rehabilitation program rather than definitive evidence of a causal treatment effect.

## Data Availability

The raw data supporting the conclusions of this article will be made available by the authors, without undue reservation.

## References

[1] Amput P Udomkichpagon P Wongphon S. Impact of duration of recovery from COVID-19 infection on physical performance in post-COVID-19 patients. COVID. (2025) 5:140. 10.3390/covid5080140

[2] Nalbandian A Sehgal K Gupta A Madhavan MV McGroder C Stevens JS. Post-acute COVID-19 syndrome. Nat Med. (2021) 27:601–15. 10.1038/s41591-021-01283-z 33753937 PMC8893149

[3] Perego E. Overview and pathophysiology of long COVID. COVID. (2026) 6:53. 10.3390/covid6030053

[4] Núñez-Cortés R Malhue-Vidal C Gath F Valdivia-Lobos G Torres-Castro R Cruz-Montecinos C. The impact of charlson comorbidity Index on the functional capacity of COVID-19 survivors: a prospective cohort study with one-year follow-up. Int J Environ Res Public Health. (2022) 19:7473. 10.3390/ijerph19127473 PMC9223623 35742722

[5] Singh I Joseph P Heerdt PM Cullinan M Lutchmansingh DD Gulati M. Persistent exertional intolerance after COVID-19. Chest. (2022) 161:54–63. 10.1016/j.chest.2021.08.010 34389297 PMC8354807

[6] Cornwell WK III Levine BD Baptiste D Bhave N Desai S Dineen E. Intolerância ao Exercício e Resposta ao Treinamento em Pacientes com Sequelas Pós-Acutas de SARS-CoV2 (Long COVID): Uma Declaração Científica da American Heart Association. Circulación. (2025) 152(5):e50–62. 10.1161/CIR.0000000000001348 40583757

[7] Dorelli G Sartori G Fasoli G Ridella N Bianchini N Braggio M. Persisting exercise ventilatory inefficiency in subjects recovering from COVID-19. Longitudinal data analysis 34 months post-discharge. BMC Pulm Med. (2024) 24:258. 10.1186/s12890-024-03070-1 38796432 PMC11128102

[8] Koleničová V Vňuková MS Anders M Fišerová M Raboch J Ptáček R. A review article on exercise intolerance in long COVID: unmasking the causes and optimizing treatment strategies. Med Sci Monit. (2023) 29::e941079-1–e941079-9. 10.12659/MSM.941079 PMC10619330 37897034

[9] Taketa T Uchiyama Y Miyagi Y Yamakawa S Seo T Yanagida A. Long-term health-related quality of life and physical function of COVID-19 survivors with ICU-acquired weakness. Prog Rehabil Med. (2024) 9:20240012. 10.2490/prm.20240012 38572470 PMC10985412

[10] Gonzalez A Abrigo J Achiardi O Simon F Cabello-Verrugio C. Intensive care unit-acquired weakness: a review from molecular mechanisms to its impact in COVID-2019. Eur J Transl Myol. (2022) 32:10511. 10.4081/ejtm.2022.10511 36036350 PMC9580540

[11] Oliveira MC Alves LR Soares JMP Souza SKA Silva BMR Fonseca AL. Health-Related quality of life and functional Status of post-COVID-19 patients. Int J Environ Res Public Health. (2025) 22(338):338. 10.3390/ijerph22030338 40238313 PMC11941946

[12] Mo X Jian W Su Z Chen M Peng H Peng P. Abnormal pulmonary function in COVID-19 patients at time of hospital discharge. Eur Respir J. (2020) 55:2001217. 10.1183/13993003.01217-2020 32381497 PMC7236826

[13] Pleguezuelos E Del Carmen A Moreno E Ortega P Robles A Serra-Prat M. Impaired pulmonary and muscle function during moderate exercise in female patients recovered from SARS-CoV-2. Sci Rep. (2022) 12:20943. 10.1038/s41598-022-24941-9 36464697 PMC9719894

[14] Ferreira EVM Oliveira RKF. Mechanisms of exercise intolerance after COVID-19: new perspectives beyond physical deconditioning. J Bras Pneumol. (2021) 47:e20210406. 10.36416/1806-3756/e20210406 34878057 PMC9013534

[15] Mayer KP Ismaeel A Kalema AG Montgomery-Yates AA Soper MK Kern PA. Persistent fatigue, weakness, and aberrant muscle mitochondria in survivors of critical COVID-19. Crit Care Explor. (2024) 6:e1164. 10.1097/CCE.0000000000001164 39412208 PMC11487221

[16] Nóbrega Júnior JC Brandão D Xavier D Torres R Soares Brandão S Formiga M. Respiratory rehabilitation after COVID-19: efficacy of inspiratory muscle training on lung function, quality of life and sleep quality: a randomized clinical trial. COVID. (2026) 6(22):22. 10.3390/covid6010022

[17] Volckaerts T Ruttens D Quadflieg K Burtin C Cops D De Soomer K. Improved functional exercise capacity after primary care pulmonary rehabilitation in patients with long COVID (PuRe-COVID): a pragmatic randomised controlled trial. BMJ Open Respir Res. (2025) 12:e003653. 10.1136/bmjresp-2025-003653 41253410 PMC12636949

[18] Spruit MA Singh SJ Garvey C ZuWallack R Nici L Rochester C. An official American thoracic society/European respiratory society statement: key concepts and advances in pulmonary rehabilitation. Am J Respir Crit Care Med. (2013) 188:e13–64. 10.1164/rccm.201309-1634ST 24127811

[19] Des Jarlais DC Lyles C Crepaz N Group TREND. Improving the reporting quality of nonrandomized evaluations of behavioral and public health interventions: the TREND statement. Am J Public Health. (2004) 94:361–6. 10.2105/AJPH.94.3.361 14998794 PMC1448256

[20] Holland AE Spruit MA Troosters T Puhan MA Pepin V Saey D. An official European respiratory society/American thoracic society technical standard: field walking tests in chronic respiratory disease. Eur Respir J. (2014) 44:1428–46. 10.1183/09031936.00150314 25359355

[21] Britto RR Probst VS Andrade AFD Samora GAR Hernandes NA Marinho PEM. Reference equations for the six-minute walk distance based on a Brazilian multicenter study. Braz J Phys Ther. (2013) 17:556–63. 10.1590/S1413-35552012005000122 24271092 PMC4207140

[22] Krupp LB LaRocca NG Muir-Nash J Steinberg AD. The fatigue severity scale. Application to patients with multiple sclerosis and systemic lupus erythematosus. Arch Neurol. (1989) 46:1121–3. 10.1001/archneur.1989.00520460115022 2803071

[23] Bestall JC Paul EA Garrod R Garnham R Jones PW Wedzicha JA. Usefulness of the medical research council (MRC) dyspnoea scale as a measure of disability in patients with chronic obstructive pulmonary disease. Thorax. (1999) 54:581–6. 10.1136/thx.54.7.581 10377201 PMC1745516

[24] Machado FVC Meys R Delbressine JM Vaes AW Goërtz YMJ Van Herck M. Construct validity of the post-COVID-19 functional Status scale in adult subjects with COVID-19. Health Qual Life Outcomes. (2021) 19:40. 10.1186/s12955-021-01691-2 33536042 PMC7856622

[25] Klok FA Boon GJAM Barco S Endres M Geelhoed JJM Knauss S. The post-COVID-19 functional Status scale: a tool to measure functional status over time after COVID-19. Eur Respir J. (2020) 56:2001494. 10.1183/13993003.01494-2020 32398306 PMC7236834

[26] Pauwels RA Buist AS Calverley PM Jenkins CR Hurd SS, GOLD Scientific Committee. Global strategy for the diagnosis, management, and prevention of chronic obstructive pulmonary disease. NHLBI/WHO global initiative for chronic obstructive lung disease (GOLD) workshop summary. Am J Respir Crit Care Med. (2001) 163:1256–76. 10.1164/ajrccm.163.5.2101039 11316667

[27] Garber CE Blissmer B Deschenes MR Franklin BA Lamonte MJ Lee I-M. Quantity and quality of exercise for developing and maintaining cardiorespiratory, musculoskeletal, and neuromotor fitness in apparently healthy adults: guidance for prescribing exercise. Med Sci Sports Exerc. (2011) 43:1334–59. 10.1249/MSS.0b013e318213fefb 21694556

[28] Zainuldin R Mackey MG Alison JA. Prescription of walking exercise intensity from the 6-minute walk test in people with chronic obstructive pulmonary disease. J Cardiopulm Rehabil Prev. (2015) 35:65–9. 10.1097/HCR.0000000000000074 24983708

[29] American College of Sports Medicine. American College of Sports Medicine position stand. Progression models in resistance training for healthy adults. Med Sci Sports Exerc. (2009) 41:687–708. 10.1249/MSS.0b013e3181915670 19204579

[30] Paluch AE Boyer WR Franklin BA Laddu D Lobelo F Lee D- Resistance exercise training in individuals with and without cardiovascular disease: 2023 update: a scientific statement from the American Heart Association. Circulation. (2024) 149:e217–31. 10.1161/CIR.0000000000001189 38059362 PMC11209834

[31] Grgic J Lazinica B Schoenfeld BJ Pedisic Z. Test-Retest reliability of the one-repetition maximum (1RM) strength assessment: a systematic review. Sports Med Open. (2020) 6(1):31. 10.1186/s40798-020-00260-z 32681399 PMC7367986

[32] Nyberg A Saey D Martin M Maltais F. Acute effects of low-load/high-repetition single-limb resistance training in COPD. Med Sci Sports Exerc. (2016) 48:2353–61. 10.1249/MSS.0000000000001027 27337174

[33] Hockele LF Sachet Affonso JV Rossi D Eibel B. Pulmonary and functional rehabilitation improves functional capacity, pulmonary function and respiratory muscle strength in post COVID-19 patients: pilot clinical trial. Int J Environ Res Public Health. (2022) 19:14899. 10.3390/ijerph192214899 36429613 PMC9691070

[34] Faul F Erdfelder E Buchner A Lang A-G. Statistical power analyses using G*power 3.1: tests for correlation and regression analyses. Behav Res Methods. (2009) 41:1149–60. 10.3758/BRM.41.4.1149 19897823

[35] Faul F Erdfelder E Lang A-G Buchner A. G*power 3: a flexible statistical power analysis program for the social, behavioral, and biomedical sciences. Behav Res Methods. (2007) 39:175–91. 10.3758/BF03193146 17695343

[36] Gloeckl R Leitl D Jarosch I Schneeberger T Nell C Stenzel N. Benefits of pulmonary rehabilitation in COVID-19: a prospective observational cohort study. ERJ Open Res. (2021) 7:00108-02021. 10.1183/23120541.00108-2021 34095290 PMC7957293

[37] Stavrou VT Tourlakopoulos KN Vavougios GD Papayianni E Kiribesi K Maggoutas S. Eight weeks unsupervised pulmonary rehabilitation in previously hospitalized of SARS-CoV-2 infection. J Pers Med. (2021) 11:806. 10.3390/jpm11080806 34442450 PMC8399744

[38] Oliveira MC Carrijo MM Afonso JP Moura RS Oliveira LF Fonseca AL. Effects of hospitalization on functional status and health-related quality of life of patients with COVID-19 complications: a literature review. Man Ther. Posturol Rehabil J. (2022) 20. 10.17784/mtprehabjournal.2022.20.1239

[39] Gesser AF Campos ML Artismo RS Karloh M Matte DL. Impact of COVID-19 critical illness on functional status, fatigue symptoms, and health-related quality of life one-year after hospital discharge: a systematic review and meta-analysis. Disabil Rehabil. (2024) 46:4086–97. 10.1080/09638288.2023.2266365 37818936

[40] Magdy DM Metwally A Tawab DA Hassan SA Makboul M Farghaly S. Long-term COVID-19 effects on pulmonary function, exercise capacity, and health status. Ann Thorac Med. (2022) 17:28–36. 10.4103/atm.atm_82_21 35198046 PMC8809128

[41] Volpato E Poletti V De Candia ML Palma L Pilon A Carpagnano GE. Breathing under pressure: psychological burden and recovery trajectories in patients receiving non-invasive respiratory support from acute COVID-19 to respiratory rehabilitation. Med Sci (Basel). (2026) 14:270. 10.3390/medsci14020270 42201062 PMC13214929

[42] Carrijo MM Oliveira MC Canedo WAO Afonso JPR Paixão HNC Alves LR. Post-Traumatic stress disorder, anxiety, and depression in post-COVID-19 patients undergoing psychotherapy: a nonrandomized clinical trial. COVID. (2025) 5:184. 10.3390/covid5110184

[43] Li S Dai B Hou Y Zhang L Liu J Hou H. Effect of pulmonary rehabilitation for patients with long COVID-19: a systematic review and meta-analysis of randomized controlled trials. Ther Adv Respir Dis. (2025) 19:17534666251323482. 10.1177/17534666251323482 40083165 PMC11907626

[44] Oliveira MR Hoffman M Jones AW Holland AE Borghi-Silva A. Effect of pulmonary rehabilitation on exercise capacity, dyspnea, fatigue, and peripheral muscle strength in patients with post-COVID-19 syndrome: a systematic review and meta-analysis. Arch Phys Med Rehabil. (2024) 105:1559–70. 10.1016/j.apmr.2024.01.007 38311096

[45] Nopp S Moik F Klok FA Gattinger D Petrovic M Vonbank K. Outpatient pulmonary rehabilitation in patients with long COVID improves exercise capacity, functional Status, dyspnea, fatigue, and quality of life. Respiration. (2022) 101:593–601. 10.1159/000522118 35203084 PMC9059007

[46] Bohannon RW Crouch R. Minimal clinically important difference for change in six-minute walk test distance of adults with pathology: a systematic review. J Eval Clin Pract. (2017) 23:377–81. 10.1111/jep.12629 27592691

[47] Pinto EF Albuquerque Filho NJB Leite JC Gusmão TME De Souza LN Silva Júnior RR. Exercise-Based rehabilitation in severe COVID-19 survivors with long COVID: a randomized controlled pilot study. Med Sci (Basel). (2026) 14:222. 10.3390/medsci14020222 42201014 PMC13214807

[48] Leite F Santos Silva A Ferreira S Brito C Leite Â. ICU Admission and post-discharge mortality in COVID-19: different risk factors across clinical phases. Med Sci (Basel). (2026) 14:255. 10.3390/medsci14020255 42201047 PMC13214800

[49] Kolck J Hosse C Fehrenbach U Beetz NL Auer TA Pille C. Disease entity impacts muscle wasting in the ICU with COVID-19 patients losing muscle nearly twice as fast. Sci Rep. (2025) 15:20176. 10.1038/s41598-025-05912-2 40542071 PMC12181421

[50] Santana PV Caruso P Roberto Ribeiro de Carvalho C. Persistent dyspnea in COVID-19 survivors without structural cardiopulmonary disease: multidimensional phenotyping at 4 years. Ann Am Thorac Soc. (2026):aaoag126. 10.1093/annalsats/aaoag126 [Epub ahead of print]. 42161351

[51] Floridia M Weimer LE Lo Forte A Palange P Agostoni P Ciardi MR. Dyspnea and fatigue in long-COVID: definition of risk factors and of DLCO-based phenotypes in a multicenter study of 765 patients from Italy. Respir Med. (2026) 258:108880. 10.1016/j.rmed.2026.108880 42107843

[52] Santus P Tursi F Croce G Di Simone C Frassanito F Gaboardi P. Changes in quality of life and dyspnoea after hospitalization in COVID-19 patients discharged at home. Multidiscip Respir Med. (2020) 15(1):713. 10.4081/mrm.2020.713 33117535 PMC7582021

[53] Fonseca AL Oliveira MC Fonseca DRP Afonso JPR Paixão HNC Júnior JBSR. Heart rate recovery after six-Minute walk test, pulmonary function, dyspnea, and functional Status after COVID-19. COVID. (2026) 6:82. 10.3390/covid6050082

[54] Neto MG Suzart Coutinho de Araujo W Pinto ACPN Saquetto MB Martinez BP Gomes VA. Effects of physical rehabilitation interventions on exercise performance, dyspnea, and health-related quality of life in acute and post-acute COVID-19 patients: systematic review and meta-analysis. Chronic Illn. (2025) 21:183–204. 10.1177/17423953241306275 40017108

[55] Ji G Chen C Zhou M Wen W Wang C Tang J. Post-COVID-19 fatigue among COVID-19 in patients discharged from hospital: a meta-analysis. J Infect. (2022) 84:722–46. 10.1016/j.jinf.2022.01.030 35101536 PMC8801976

[56] Poole-Wright K Guennouni I Sterry O Evans RA Gaughran F Chalder T. Fatigue outcomes following COVID-19: a systematic review and meta-analysis. BMJ Open. (2023) 13:e063969. 10.1136/bmjopen-2022-063969 37185637 PMC10151247

[57] Rooney S McFadyen A Wood L Moffat F Paul L. Minimally important difference of the fatigue severity scale and modified fatigue impact scale in people with multiple sclerosis. Mult Scler Relat Disord. (2019) 35:158–63. 10.1016/j.msard.2019.07.028 31400557

[58] Pérez-Gisbert L Brea-Gómez B Valenza MC Calvache-Mateo A Ortiz-Rubio A Torres-Sánchez I. Does pulmonary rehabilitation improve fatigue in patients with post-COVID-19 syndrome? A meta-analysis of randomized clinical trials. Disabil Rehabil. (2026) 48:646–66. 10.1080/09638288.2025.2546553 40827836

[59] Gomes dos Santos EG Vieira da Costa K Cordeiro de Souza IT Victor dos Santos Felix J Furtado Brandão CB Michelle de Souza Fernandes V. Effects of a cardiopulmonary rehabilitation protocol on functional capacity, dyspnea, fatigue, and body composition in individuals with post-COVID-19 syndrome: a randomized controlled trial. Physiother Res Int. (2024) 29:e2086. 10.1002/pri.2086 38572991

[60] Martins RL Monteiro EDSS De Lima AMJ Santos AC Brasileiro-Santos MS. Effect of telerehabilitation on pulmonary function, functional capacity, physical fitness, dyspnea, fatigue, and quality of life in COVID-19 patients: a systematic review and metanalysis. Telemed J E Health. (2024) 30:e2256–86. 10.1089/tmj.2023.0653 38920003

[61] Simonelli C Paneroni M Vitacca M Ambrosino N. Measures of physical performance in COVID-19 patients: a mapping review. Pulmonology. (2021) 27:518–28. 10.1016/j.pulmoe.2021.06.005 34284976 PMC8221906

[62] de Jong CMM Van Raaij BFM Antoni L(ML) Arbous S(MS) Geelhoed M(JJM) De Graaf MA. Evaluation of the post-COVID-19 functional Status scale based on its use during a one-year follow-up of COVID-19 survivors. COVID. (2026) 6:81. 10.3390/covid6050081

[63] Taboada M Moreno E Cariñena A Rey T Pita-Romero R Leal S. Quality of life, functional status, and persistent symptoms after intensive care of COVID-19 patients. Br J Anaesth. (2021) 126:e110–3. 10.1016/j.bja.2020.12.007 33413976 PMC7833644

[64] Mohamed Hussein AAR Saad M Zayan HE Abdelsayed M Moustafa M Ezzat AR. Post-COVID-19 functional status: relation to age, smoking, hospitalization, and previous comorbidities. Ann Thorac Med. (2021) 16:260–5. 10.4103/atm.atm_606_20 34484441 PMC8388571

[65] Halaweh H Ghannam I. The devastating trio of sarcopenia, frailty, and COVID-19—a systematic review and meta-analysis. Clin Nutr ESPEN. (2022) 51:143–51. 10.1016/j.clnesp.2022.07.015 36184198 PMC9356632

[66] Piotrowicz K Ryś M Perera I Gryglewska B Fedyk-Łukasik M Michel J-P. Factors associated with mortality in hospitalised, non-severe, older COVID-19 patients—the role of sarcopenia and frailty assessment. BMC Geriatr. (2022) 22(1):941. 10.1186/s12877-022-03571-w 36476473 PMC9727948

[67] Piotrowicz K Gąsowski J Michel J-P Veronese N. Post-COVID-19 acute sarcopenia: physiopathology and management. Aging Clin Exp Res. (2021) 33:2887–98. 10.1007/s40520-021-01942-8 34328636 PMC8323089

[68] Cecchetto A Guarnieri G Torreggiani G Vianello A Baroni G Palermo C. Dyspnea in post-acute COVID-19: a multi-parametric cardiopulmonary evaluation. J Clin Med. (2023) 12:4658. 10.3390/jcm12144658 37510773 PMC10380208

[69] Morgan S Smith JM Thomas B Moreno M Visovsky C Beckie T. Risk factors and predictors for persistent dyspnea post-COVID-19: a systematic review. Clin Nurs Res. (2025) 34:195–212. 10.1177/10547738251314076 39876047

[70] Ramírez-Vélez R Oscoz-Ochandorena S García-Alonso Y García-Alonso N Legarra-Gorgoñon G Oteiza J. Maximal oxidative capacity during exercise is associated with muscle power output in patients with long coronavirus disease 2019 (COVID-19) syndrome. A moderation analysis. Clin Nutr ESPEN. (2023) 58:253–62. 10.1016/j.clnesp.2023.10.009 38057014

[71] Yang L-L Yang T. Pulmonary rehabilitation for patients with coronavirus disease 2019 (COVID-19). Chronic Dis Transl Med. (2020) 6:79–86. 10.1016/j.cdtm.2020.05.002 32411496 PMC7221406

